# Refocusing graduate gross anatomy training: Curating future content experts

**DOI:** 10.1002/ase.2535

**Published:** 2024-11-17

**Authors:** Gail Elliott, Grace Pinhal‐Enfield

**Affiliations:** ^1^ Department of Neuroscience and Cell Biology, Office of Education, Robert Wood Johnson Medical School Rutgers University Piscataway New Jersey USA; ^2^ Department of Neuroscience and Cell Biology, Anatomical Association, Robert Wood Johnson Medical School Rutgers University Piscataway New Jersey USA

**Keywords:** graduate anatomy education, learning styles, spaced repetition testing, team‐based learning

## Abstract

Graduate anatomy courses should be designed based on several needs. These include preparation for how to study in medical school and other healthcare programs, integrating multiple ways of engaging with the material, including repetition for long‐term retention, and training of anatomy educators. Our graduate anatomy course presents an example of a balanced course structure that caters to the varying needs of different learners and encourages interest in anatomy education as a profession. By refocusing graduate gross anatomy training, we aim to support learners pursuing fields that utilize anatomy, including healthcare professions as well as future content experts to address the shortage of qualified anatomy educators.

## BACKGROUND

Pre‐professional school courses in anatomy are designed to appeal to students seeking to strengthen their medical school applications.^1^ Some of these pre‐professional courses imitate the pace and content of medical courses, while potentially doing a disservice to students' learning experience. This work proposes an approach to graduate anatomy education focused on engaging learners to pursue multiple career pathways.^1^ In contrast to complete anatomical study with full body dissection, graduate anatomy courses that focus on specific regions afford time for learners to explore the subject through various didactic approaches that offer different angles of the same topic.

Anatomy courses provide students with a foundational knowledge for medical school or other healthcare programs. We propose a format that teaches anatomy within the Master of Biomedical Sciences, School of Graduate Studies, Rutgers University, in addition to developing effective study practices and implementing multiple ways of engaging with the materials. Recent course development has sought to meet fewer objectives on anatomy, while increasing depth of study using different educational formats. This offers a greater exploration of specific topics, while providing skill sets for learners interested in pursuing multiple different career pathways. Modifications like these encourage inclusion of students with varied future healthcare professions and may hold the key to addressing critical shortages in anatomy content experts, and should not be overlooked.^2^, ^3^

This manuscript seeks to offer an example of a balanced course structure that is beneficial to students across the spectrum of capability for their understanding and enjoyment of anatomy. It also intends to curate interest at all levels with the hope of encouraging consideration of anatomy education as a profession through enjoyment of the subject.

## ACTIVITY

In the master's in biomedical sciences graduate program, the 13‐week anatomy course holds classes on Tuesday and Thursday afternoons during the spring semester. Most of these students come into the program to improve their grades in the hopes of applying to medical schools during the next cycle. Therefore, the program incorporates some gross anatomy to get a start on the depth of anatomy taught in medical schools, while also helping students to address their study habits through meetings with our learning skills departments, deans, and faculty. These are students who are building their knowledge base in basic sciences to support their professional school applications.

The majority of this student body take the course with an interest in boosting their academic profile, and the course is focused on meeting them there. Therefore, the aim is to provide insight into a professional‐level course, while ensuring development of the skills necessary to study at this level. Successful graduates of this course potentially have an advantage in approaching the volume and types of material, activities, and assessments at a professional level.

### Original course format

The program's original course took the form of traditional medical school anatomy courses, with didactic lectures, followed by related dissection laboratories (see Table 1). Topics covered included anatomical terminology, back, spinal cord, upper limb, thorax, and abdomen.

**TABLE 1:** The outline of the original 13‐week graduate gross anatomy course is shown below.

Week	Tuesday	Thursday	Friday
1		Course Introduction and Anatomical Terminology (1 h lecture)	
		Gross Anatomy Lab Introduction (1 h lecture)	
2	Joint & Muscle Action (1.5 h lecture)	Circulatory System (1 h lecture)	
3	Overview of the Nervous System (2 h lecture)	Herniated Nucleus Pulposus and Imaging of the Back (1 h lecture)	
4	Back, Spinal Cord, and Posterior Shoulder (2 h lecture)	Back, Spinal Cord, and Posterior Shoulder (3 h lab)	TA Lab Review Session (1.5 h lab review)
5	Pectoral Region, Shoulder, and Axilla (2 h lecture)	Pectoral Region, Axilla, and Anterior Arm (3 h lab)	Lab Review Session (1.5 h lab review)
6	Arm, Forearm, and Hand (2 h lecture)	Forearm and Hand (3 h lab)	TA Lab Review Session (1.5 h lab review)
7	Upper Limb Nerve Injuries (2 h lecture)	Faculty Lab Review Session (3 h lab review)	
8	Practical Exam 1	Individual MCQ Exam 1	
9	Functional Anatomy of the Adult Heart (2 h lecture)	Lungs, Pleura, and Mechanics of Respiration (2 h lecture)	
		Thoracic Wall, Pleura, and Lungs (3 h lab)	TA Lab Review Session (1.5 h lab review)
10	Anterior Abdominal Wall and Inguinal Region (2 h lecture)	Anterior Abdominal Wall, Inguinal Region, and Foregut (3 h lab)	TA Lab Review Session (1.5 h lab review)
	Autonomic Nervous System (0.5 h podcast)		
11	Abdominal Cavity, Mesentery, and Viscera (1 h lecture)	Midgut, Hindgut, and Posterior Abdominal Wall (3 h lab)	TA Lab Review Session (1.5 h lab review)
	Abdominal Circulation and Innervation (1 h lecture)		
12	Kidney and Reproductive Viscera (2 h lecture)	Faculty Lab Review Session (3 h lab review)	
	Abdominal Imaging (1 h podcast)		
13	Practical Exam 2	Individual MCQ Exam 2	

Final grades were calculated based on two practical examinations and two individual multiple‐choice examinations. The cohort is mostly composed of pre‐med students, and the course was initially designed to boost grades for medical school applications. However, grades for the ~90 students typically separated into two groups, one showing mastery of material, while the other struggling to pass with a C+ overall (77%–79%). From an educator's perspective, it was disheartening to see more than half of the cohort become despondent and ultimately meant that the course was unsuitable for all learners. This situation highlights that there was a mismatch between the course structure and the diverse learning styles of the students.

Course performance evaluations made it clear that a dramatic change was necessary to support different types of learners. As a result, the course was refocused to create an approach enabling students to study effectively and develop study techniques that would promote success and life‐long learning. These changes were developed to encourage effective study habits, exam‐taking strategies, and time management, which may be helpful to students working full‐time during course efforts toward their degree.

### Updated course format

The program's updated graduate anatomy course includes the changes described below and listed in Table 2.

**TABLE 2:** The outline of the 13‐week updated course is shown below.

Week	Tuesday	Thursday	Friday	
1	Course Introduction (1 h lecture)	Anatomical Terminology (1 h lecture)	Study and Exam‐Taking Tips and Strategies (1 h podcast)	
	Virtual Anatomy Lab Introduction (1 h podcast)	Diagnostic Imaging (1 h podcast)		
2	Circulatory System (2 h lecture)	Thoracic Wall, Pleura, and Lungs (3 h lab)	TA Lab Review Session (1.5 h lab review)	Progress Quiz 1 (2% of overall grade)
3	Lungs, Pleura, and Mechanics of Ventilation (2 h lecture)	Superior, Middle, and Posterior Mediastinum (3 h lab)	TA Lab Review Session (1.5 h lab review)	
4	Functional Anatomy of the Heart (2 h lecture)	TBL 1: Case study (adult heart & embryology) (2 h)	TA Lab Review Session (1.5 h lab review)	Progress Quiz 2 (2% of overall grade)
5	Overview of the Nervous System (1.5 h lecture)	Back and Spinal Cord (3 h lab)	TA Lab Review Session (1.5 h lab review)	
6	Back, Posterior Shoulder, and Spinal Cord (2 h lecture)	TBL 2: Understanding the Somatic and Autonomic Nervous Systems (2 h TBL)	TA Lab Review Session (1.5 h lab review)	Progress Quiz 3 (2% of overall grade)
7	Practical Exam 1 (2 h)	Individual and Team MCQ Exams 1 (4 h)		
8	Herniated Disc, Nucleus Pulposus, and Imaging of the Back (1 h lecture)	Pectoral Region, Axilla, and Brachial Plexus (3 h lab)	TA Lab Review Session (1.5 h lab review session)	
	Joints and Muscle Action (1 h lecture)			
9	Pectoral Region, Axilla, and Brachial Plexus (2 h lecture)	TBL 3: Diagnostic Imaging and the Herniated Disc (2 h TBL)	TA Lab review session (1.5 h lab review)	Progress Quiz 4 (2% of overall grade)
10	Compartments of the Arm and Cubital Fossa (2 h lecture)	Compartments of the Arm and Cubital Fossa (3 h lab)	TA Lab Review Session (1.5 h lab review)	
11	Forearm and Hand (2 h lecture)	Forearm and Hand (3 h lab)	TA Lab Review Session (1.5 h lab review)	
12	Upper Limb Nerve Injuries (2 h lecture)	TBL 4: Upper Limb Nerve Injuries (2 h TBL)	TA Lab Review Session (1.5 h lab review)	Progress Quiz 5 (2% of overall grade)
13	Practical Exam 2 (2 h)	Individual and Team MCQ Exams 2 (4 h)		

#### Progress quizzes

Five cumulative progress quizzes (20 questions each) were introduced every 2 weeks encompassing laboratory, lecture, and team‐based learning (TBL) content. These were implemented because research shows that frequent testing helps to identify struggling students.^4^ This spaced repetition testing continually reinforces content. Students had the opportunity to take these for a full week, it was open book and timed.

There were also 10 practice questions available under each lecture. These coupled with the iRAT (individual readiness assurance test, 10 questions) and gRAT (group readiness assurance test, 10 questions) quizzes for the TBLs created a strong bank of practice questions for student learning. However, there was no overlap between any questions during the course and the examinations (i.e., questions that appeared during the course were not used in the formative midterm and final examinations).

#### Preparation

Students were expected to pre‐read the laboratory manual and take a pre‐laboratory quiz (10 questions). A post‐laboratory quiz (10 questions) was then completed at the end of the laboratory as a group with different questions before the students left for the day. These quizzes enabled the students to prepare for the laboratory ahead and future iterations of this program will ensure the importance of these quizzes with a grade point association.

#### Laboratory first

Dissection laboratory before the lecture ensured students were familiar with structures before deepening their understanding through functionally and clinically correlated lectures. This approach had the benefit of enabling greater learner engagement during lectures and laboratory as students seemed better prepared overall with the required preparation. This was evidenced by the higher laboratory examination scores and the greater engagement with all faculty in laboratory and lecture. The greater emphasis on active learning required students to read before going into the laboratory. Introduction to the structures before the more clinically focused lecture allowed the students time to absorb some of the material before additional layers were added to it.

#### Small group activities

Group work has shown that students' attitudes and academic performance improved.^5^, ^6^ Therefore, in addition to dissection teams, the same groups worked together on TBL topics that focused on concepts students typically struggled with (e.g., the blood supply of the heart, the spinal cord cross‐section, medical imaging, and the brachial plexus and upper limb nerve injuries). Thus, teamwork was a central thread throughout the course and students made friends, cutting the isolation for those who were frequently working and not on campus, and provided channels for communication between students for questions. Activities incorporated into the TBLs frequently involved an activity using clay to build, because such exercises have been shown to have beneficial impacts on student learning.^7^, ^8^

#### Group examinations

The group examination immediately followed the individual MCQ examination. The same examination was provided to each student group, and they discussed the question before using scratch cards to identify the answer. This process allowed students to fundamentally understand where their thinking was incorrect and became an invaluable way of providing feedback.^5^, ^6^

#### Content reduction

The abdominal content was removed from the course. The remaining course content offered contrasting study of thoracic viscera with autonomic innervation and upper limb with somatic innervation. This allowed organs and the bigger picture of autonomic versus somatic innervation to be captured, while the abdomen could be covered in future study. Finally, Table 3 shows the old versus the new breakdown of grades.

**TABLE 3:** Grading breakdown for the original course versus the updated course formats are shown.

	Original course		Updated course	
	Exam	(%)	Exam	(%)
Exam 1	Practical 1	15	Practical 1	10
	Individual MCQ 1	35	Individual MCQ 1	20
			Team MCQ 1	10
Exam 2	Practical 2	10	Practical 2	10
	Individual MCQ 2	30	Individual MCQ 2	20
			Team MCQ 2	10
Other	Gross Anatomy Lab attendance and completion of pre‐lab and post‐lab quizzes	10	TBL & Gross Anatomy Lab attendance and completion of pre‐activity and post‐activity quizzes	10
			Progress Quizzes (5 × 2%)	10

## RESULTS AND DISCUSSION

Active learning enhances student engagement as learners take a more active role in their own studies. Emphasis on self‐study prior to activities requires learners to engage with material rather than passively listening, promoting student preparation for application and critical thinking.^9^, ^10^

The transformation in this course captured student attention by having learners work in teams to discuss content with peers and faculty. Collaborative hands‐on projects, such as teams building clay models of the brachial plexus followed up by student presentations, allowed students to engage with each other in a practical way. Encouraging active learning in a welcoming environment, where faculty are supportive and non‐judgmental, allows students to integrate knowledge and recognize that mistakes with immediate feedback improve understanding. Based on student examination performance, outcomes from these changes included enhanced retention and improved student performance.

Based on course feedback, students highlighted strengths of the course. Students found the course to display a cohesive, integrated narrative of content supported by a diversity of learning resources. Group‐based activities were also helpful and engaging and were a hallmark change in this course. The newly added TBLs reinforced and integrated knowledge and understanding of content from lecture and laboratory. For example, the brachial plexus was studied in the laboratory and reinforced in lecture and TBL using clay modeling of the brachial plexus. Group examinations, which followed individual examinations, also allowed students to gain immediate feedback by reviewing with peers. While these various interactive didactic methods supported student learning and retention, clinical correlations captured student interest. Additionally, a more focused set of topics gave students time to understand lectures and practice with repetitive formative quizzing. Together, these changes allowed for better pacing, reinforcement, retention, and student wellness.

It is critical to recognize that dramatic curricular changes require faculty organization, support, and communication. Transparency for students in understanding why the course was being changed, the aims, and how these goals would be reached was essential in developing and curating a positive learning environment.

Interestingly, some suggestions for improvement were requests for even more of the new course activities, such as clay modeling and practice questions. Some students did suggest switching the order to have lectures before laboratories, moving away from an emphasis on self‐directed study to guided study. However, while we recognize lecture to be a more familiar method for students, the course model was designed to move away from passive study and encourage interactive and engaged learning activities.

## AUTHOR CONTRIBUTIONS

**Gail Elliott:** Conceptualization; data curation; investigation; methodology; project administration; writing – original draft; writing – review and editing. **Grace Pinhal‐Enfield:** Conceptualization; investigation; methodology; writing – review and editing.

## CONFLICT OF INTEREST STATEMENT

The authors have no competing interests to declare that are relevant to the content of this article.
